# Dynamics of inhaled corticosteroid use are associated with asthma attacks

**DOI:** 10.1038/s41598-021-94219-z

**Published:** 2021-07-19

**Authors:** Joy Lee, Jacqueline Huvanandana, Juliet M. Foster, Helen K. Reddel, Michael J. Abramson, Cindy Thamrin, Mark Hew

**Affiliations:** 1 Allergy, Asthma and Clinical Immunology, The Alfred Hospital, Alfred Health, 55 Commercial Road, Prahran, Melbourne, VIC 3183 Australia; 2 School of Public Health & Preventive Medicine, Monash University, 553 St Kilda Road, Melbourne, VIC Australia; 3 The Woolcock Institute of Medical Research and The University of Sydney, 431 Glebe Point Road, Sydney, NSW Australia

**Keywords:** Asthma, Therapeutics

## Abstract

Inhaled corticosteroids (ICS) suppress eosinophilic airway inflammation in asthma, but patients may not adhere to prescribed use. Mean adherence—averaging total doses taken over prescribed—fails to capture many aspects of adherence. Patients with difficult-to-treat asthma underwent electronic monitoring of ICS, with data collected over 50 days. These were used to calculate entropy (H) a measure of irregular inhaler use over this period, defined in terms of transitional probabilities between different levels of adherence, further partitioned into increasing (H_inc_) or decreasing (H_dec_) adherence. Mean adherence, time between actuations (Gap_max_), and cumulative time- and dose-based variability (area-under-the-curve) were measured. Associations between adherence metrics and 6-month asthma status and attacks were assessed. Only H and H_dec_ were associated with poor baseline status and 6-month outcomes: H and H_dec_ correlated negatively with baseline quality of life (H:Spearman r_S_ = − 0·330, p = 0·019, H_dec:_r_S_ = − 0·385, p = 0·006) and symptom control (H:r_S_ = − 0·288, p = 0·041, H_dec:_ r_S_ = − 0·351, p = 0·012). H was associated with subsequent asthma attacks requiring hospitalisation (Wilcoxon Z-statistic = − 2.34, p = 0·019*)*, and H_dec_ with subsequent asthma attacks of other severities. Significant associations were maintained in multivariable analyses, except when adjusted for blood eosinophils. Entropy analysis may provide insight into adherence behavior, and guide assessment and improvement of adherence in uncontrolled asthma.

## Introduction

In asthma, regular inhaled corticosteroid (ICS) controller use suppresses eosinophilic airway inflammation and reduces airway hyperresponsiveness, reducing symptoms and protecting patients from potentially life-threatening attacks^1,2^. Asthma that remains uncontrolled despite the use of high-dose ICS-based controller is regarded as ‘difficult-to-treat’, and presents a complex clinical challenge^3^.

Medication adherence describes the extent to which patients use medication as prescribed^4,5^. In difficult-to-treat asthma, patients frequently deviate from prescribed use^6,7^. This can be tracked objectively by attaching electronic monitoring devices (EMDs) to a patient’s inhaler, recording the date and time of each actuation^8^. In future, electronic monitors may be routinely integrated into inhalers during manufacture^9^.

Clarifying the interplay between poor adherence and adverse outcomes could help improve adherence and enhance patient health. So far, work has focused on time-averaged metrics, typically mean adherence (total doses taken/total doses prescribed) and it has been difficult to demonstrate a relationship between asthma outcomes and mean adherence^10,11^. This averaged metric fails to capture potentially important variations in medication-taking behaviour, e.g. a mean adherence rate of 50% cannot distinguish between one patient consistently taking half the prescribed dose daily and another taking the full prescribed dose, but for only half the required period. Other metrics do take into account either the interval between doses, or the time above a minimum dose threshold, and some have shown a relationship to attack rates in airways diseases^10,12^. However, these strategies still only represent summative time-averaged metrics, and do not describe day-to-day deviations from regular prescribed usage.

We designed adherence metrics to capture via EMD the extent to which patients with difficult-to-treat asthma deviate from regular controller usage, by measuring the *entropy*—irregularity, or disorder—with which daily medication doses are taken. The concept of entropy is derived from information theory where it is used to quantify the ‘information’ in a process. Entropy has been previously applied to respiratory symptoms^13^, breathing patterns, and lung function^14^. We examined whether these entropy measures of adherence related to specific patient characteristics or predicted subsequent asthma-related clinical outcomes. For comparison, we also measured conventional mean adherence, time- and dose-based variability (using additional metrics reflecting missed days and incomplete doses respectively), and the duration of gaps in which patients completely forwent medication.

We hypothesised that the degree of *irregularity* of ICS controller usage may be more relevant in difficult-to-treat asthma, and better predict poor outcomes. Highly disordered medication-taking behaviour may place patients at higher clinical risk and may be associated with poorer outcomes.

## Methods

### Study participants

**Figure 1:**
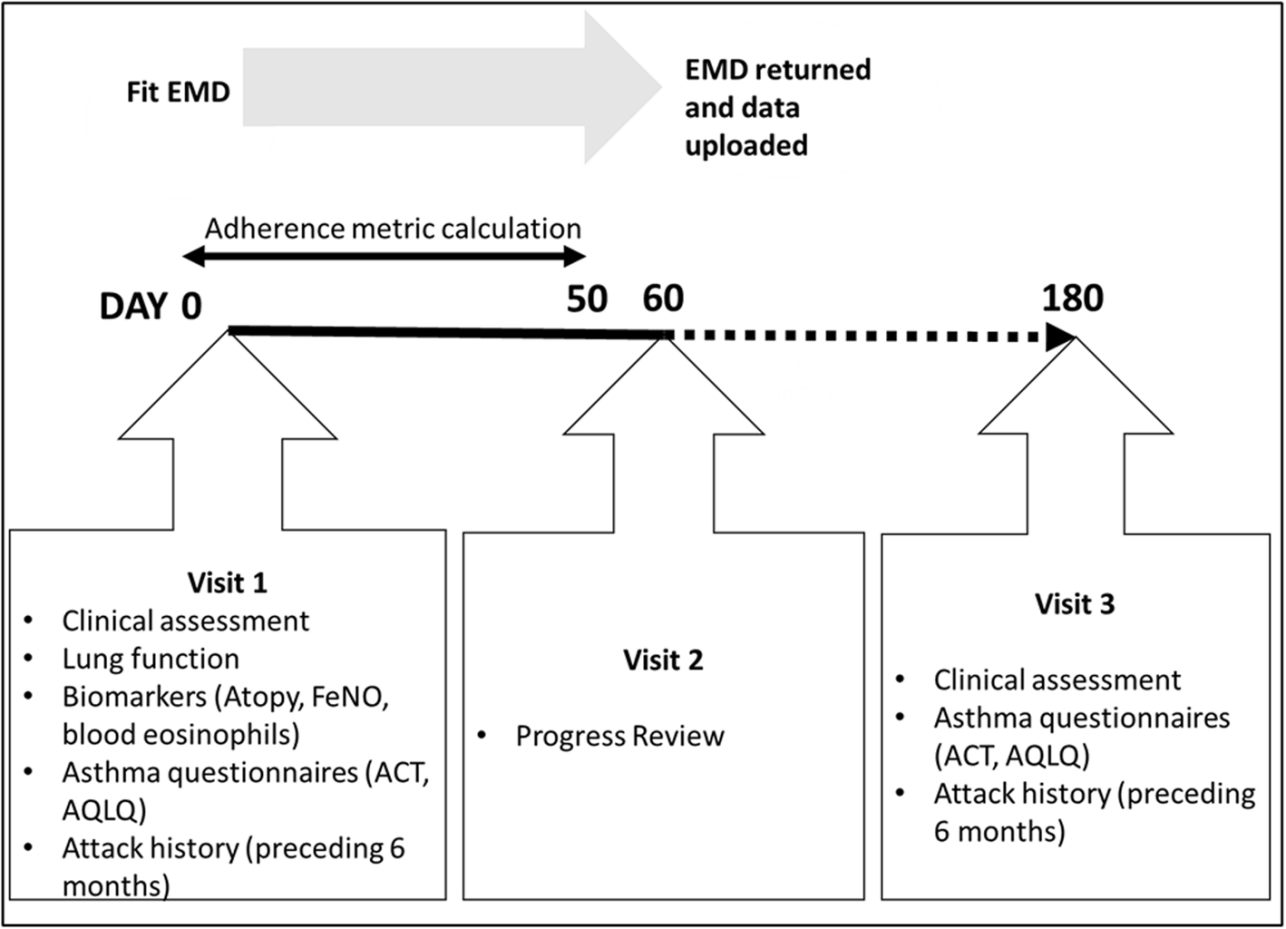
Assessment protocol timeline, visits and clinical measures. *EMD* electronic monitoring device, *ACT* asthma control test, *AQLQ* asthma quality of life. (Microsoft Powerpoint, version 2101, https://office.live.com/start/powerpoint.aspx).

Our tertiary centre receives referrals of adults with difficult-to-treat asthma from specialists in secondary care^17^. Patients underwent multidisciplinary assessment according to a pre-specified protocol over three visits over six months—previously reported in detail^18–22^—to confirm asthma diagnosis, address comorbidities, and optimise treatment (Fig. 1).

Inhaler technique was reviewed and optimised.

### Electronic devices

A compatible EMD (Adherium, Auckland, New Zealand) was fitted to the patient’s ICS-containing controller inhaler at visit one (day 0) and EMD-collected ICS data were uploaded at visit two (approximately day 60, Fig. 1). Outcomes were assessed at visit 3 (~ 180 days). EMDs were available for budesonide/formoterol (Turbuhaler and Rapihaler) and fluticasone proprionate/salmeterol (metered dose inhaler and Accuhaler). To allow uninterrupted monitoring, participants were instructed how to move the EMD if they were to change their inhaler. Audiovisual reminders were not activated during the study period but participants could access 7-day EMD data on their devices.

### Clinical outcomes

Evaluation included the Asthma Control Test^23^ and Asthma Quality of Life Questionnaire^24^ (with permission) at baseline (visit one) and 6 months (visit three) (Fig. 1). Patients were asked to recall the number of attacks in the 6 months prior to visit one, and again, in the 6-month period prior to visit three. Attacks were then confirmed by medical and prescription records where possible. Attacks were also categorised by severity, defined by worsening asthma symptoms requiring: a visit to the general practitioner (least severe); a course (or an increased dose) of oral corticosteroids (OCS; more severe); or hospitalisation (most severe). It was also noted if hospitalization required intensive care admission. Frequency of short acting bronchodilator use over the past four weeks prior to visit one were self-reported and recorded in terms of days and nights per week, as well as number of puffs per day and night.

Patients who completed three visits between August 2015 and February 2018 were eligible for study inclusion. All study protocols and data analysis were approved by the Alfred Health Ethics Committee (285/15) and the Monash University Human Research Ethics Committee (MUHREC). As data were collected as part of routine clinical care and audit, the requirement for signed informed consent was waived by the Alfred Health Ethics Committee. All methods were carried out in accordance with relevant guidelines and regulations as governed by the Australian Health Practitioner Regulation Agency (AHPRA).

### Adherence metrics

Metrics were quantified using Python (Python Software Foundation, version 3·6). For standardisation, the first 50 days of available data were extracted for each patient (Fig. 1); this excluded any days with missing data (defined as days when inhaler was not attached—logged by the device as distinct to zero adherence)*.* As the EMD was returned at visit two, adherence data were not available to day 180. Last observation carried forward was not performed to minimise the risk of introducing bias into the adherence metrics, particularly entropy.

*Entropy* (H), a measure of disorder, was adapted to the adherence data to reflect the various ways in which the patients changed their ICS-taking behavior from day to day. In information theory, a ‘Markov chain’ can be used to describe the sequence of occurrences of certain ‘states’ and the probabilities of transitioning from one state to another given the previous state (e.g. the appearance of specific sequences of letters in a message). H is then used to quantify the complexity of the information, in terms of the transitional probabilities between states, for all possible states observed. Here, we classified adherence into different levels, which are analogous to the different states of a Markov chain, similar to an approach previously applied to respiratory symptoms^13^. Given an adherence time series x, where x is the dose taken/prescribed dose expressed as a percentage, we obtained a state-based series by mapping each element of x_i_ to the state space S = {1,2,3,4,5,6,7} as follows:

**Table:** 

State, si	Dose range
1	x_i_ = 0%
2	0 < x_i_ ≤ 50%
3	50% < x_i_ < 100%
4	x_i_ = 100%
5	100 < x_i_ ≤ 200%
6	200 < x_i_ ≤ 300%
7	x_i_ > 300%

**Figure 2:**
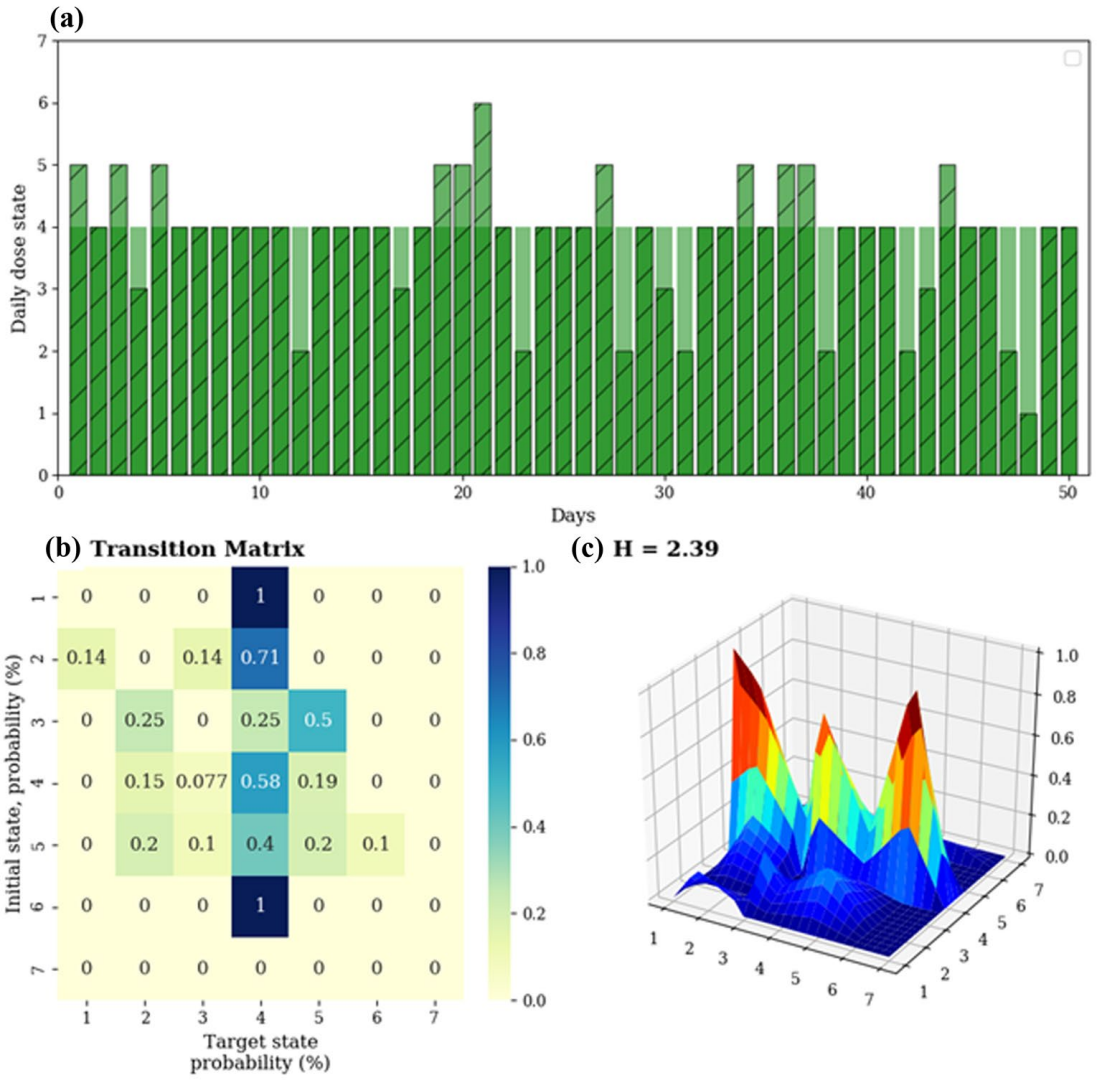
Calculation of entropy. Panel (**a**) shows a perfectly-adherent time series (green) in the background comprising 100% of prescribed puffs for 100% of the time, and an example patient time series (blue) overlaid atop the perfectly-adherent series, with instances of under- and over-adherence, both mapped to the state-based series. Panel (**b**) displays the corresponding transitional probability matrix, while panel (**c**) allows us to visualise the same matrix (and the “disorder”) in a 3-dimensional graph. The entropy of the transitional probability matrix is then calculated as $\sum_{i,j=0}^{N-1}{P}_{i,j}(-\mathrm{ln}({P}_{i,j}))$. (Python Software Foundation, version 3·6 http://www.python.org).

We then computed the 7 × 7 transitional probability matrix, which comprised probabilities *P*_*i,j*_ denoting the probability of transitioning from state *j*, given an initial state *i*, for every combination of states *i,j*. The entropy (H) of the system was determined as $H= \sum_{i,j=0}^{N-1}{P}_{i,j}(-\mathrm{ln}({P}_{i,j}))$, representing the disorder of transitions between daily dose states. An example of H calculation is shown in Fig. 2.

We further partitioned the probability matrix into “increasing” (H_inc_) or “decreasing” (H_dec_) adherence, by only considering transitions that moved from lower to higher adherence states on the next available day, or vice versa, respectively, i.e. splitting the transitional probabilities along the diagonal of the matrix. H_inc_ was determined as $\sum_{i,j=0}^{N-1}{P}_{i,j}(-\mathrm{ln}({P}_{i,j}))$, where *i* < *j*. Similarly, H_dec_ was defined as $\sum_{i,j=0}^{N-1}{P}_{i,j}(-\mathrm{ln}({P}_{i,j}))$, where *i* > *j*. Thus, while H represents the day to day changes in adherence levels in general, H_inc_ represents the different ways in which a patient may increase their adherence level, and conversely H_dec_ the different ways in which decreases in adherence may occur.

**Figure 3:**
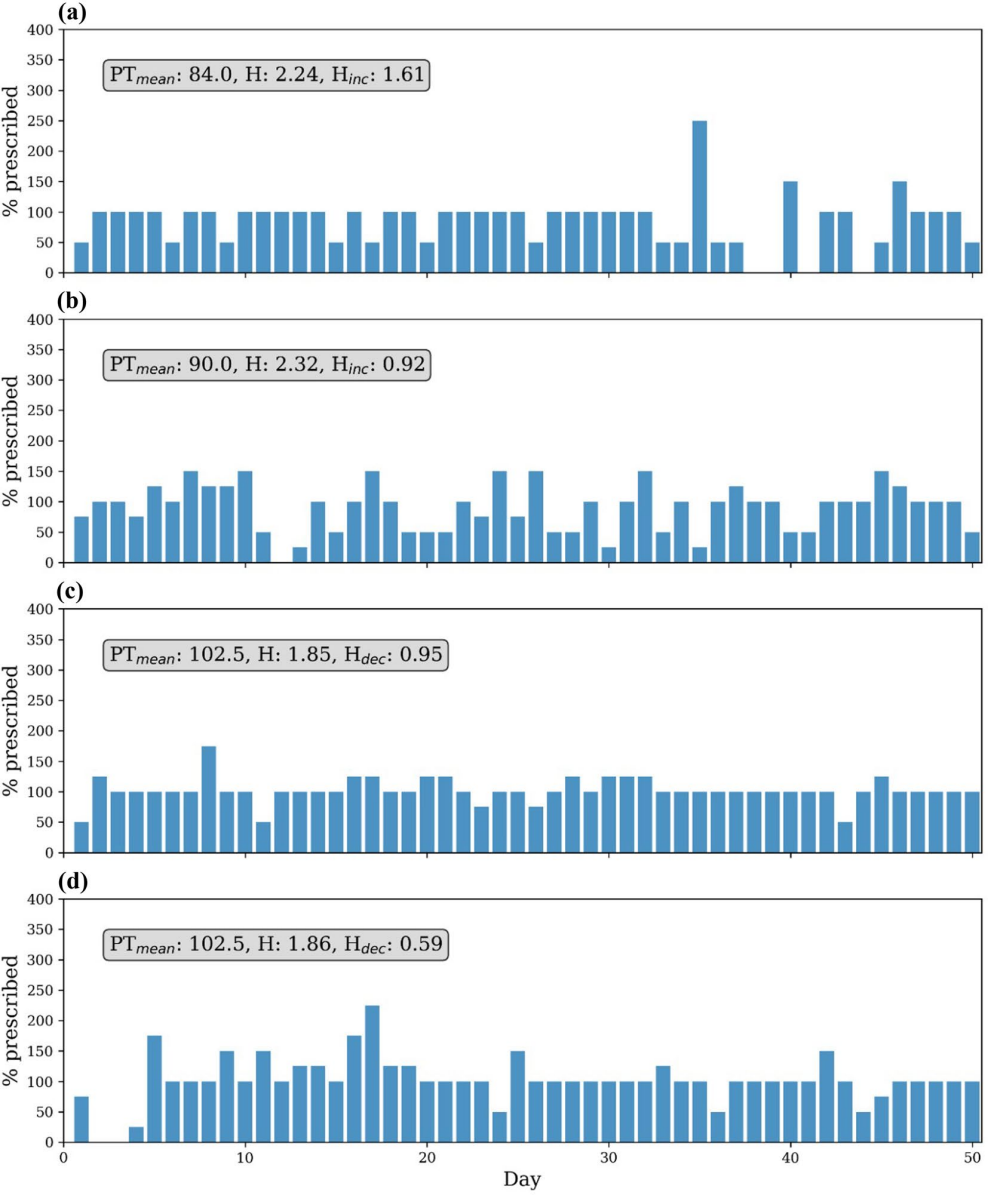
Sample adherence time series from 4 different patients over the study period. All patients had ‘good adherence’ as defined by mean adherence PT_mean_ (note not capped at 100% for the purpose of demonstrating variability), however with different increasing and decreasing entropy (H_inc_ and H_dec_) measures, which better reflect the variability in patient inhaler adherence behaviour. Panel (**a**) demonstrates a patient who took their inhaled controller on average 84% of prescribed doses with calculated entropy 2.24 and increasing entropy 1.61. Panel (**b**) demonstrates a second patient who took their controller 90% of prescribed doses, with calculated entropy of 2.32 and increasing entropy of 0.92. Panel (**c**) demonstrates a third patient who took their controller 102.5% of prescribe doses, with calculated entropy of 1.85 and decreasing entropy of 0.95. Panel (**d**) demonstrates a fourth patient who took their controller 102.5% of prescribed doses, with calculated entropy of 1.86 and decreasing entropy of 0.59. (Python Software Foundation, version 3·6 http://www.python.org).

Figure 3 illustrates how different adherence time series with the same conventional mean adherence (PT_mean_, see below) may vary in H_inc_ and H_dec_.

Conventional *mean adherence* was described as PT_mean_, expressed as a percentage of the prescribed number of puffs per day. This was capped at 100%—all higher daily instances were converted to 100% to allow comparison of the mean (PT_mean,cap_) with other published studies. PT_SD_ and PT_CV_ represented the standard deviation and coefficient of variation of PT_mean_ respectively, based on uncapped data to capture the full variability in adherence.

*Area under the curve (AUC) measures* were inspired by methods previously described^10^ to investigate both time- and dose-based adherence variability. In brief, perfect time adherence was first defined as medication taken daily (over the first 50 days), regardless of dosage. The time adherence curve was then defined as the cumulative sum of every day when medication was taken. Thus, perfect time adherence corresponded to a straight line with an area under the curve normalised to 100%. The time-based AUC (T-AUC) for an individual patient was taken as the difference between their time adherence curve and the perfect curve, expressed as a percentage deviation from 100%. In this way, the T-AUC described how consistently the patient took any medication over time. The use of the cumulative sum meant that earlier and/or larger gaps have greater effects on the T-AUC. Similarly, the dose-based area AUC (D-AUC) described the cumulative deviation from the patient’s total prescribed dose over the same 50-day period. We also multiplied the time-based deviation and the dose-based adherence for each day, to construct a composite curve. The Prod-AUC was then calculated as the cumulative deviation from the product of the perfect time x dose curves. This metric thus reflects adherence behavior in terms of both time and doses taken over the given time period.

The Gap_max_ metric described the maximum length of gaps between days when medication was last taken, regardless of number of puffs within a day, during the 50-day period.

### Statistical analysis

Relationships between adherence metrics and clinical characteristics (at baseline) and asthma outcomes (at/over six months) were examined using Spearman rank correlation (r_s_) for continuous variables, and Wilcoxon rank sum or Kruskal–Wallis tests for comparisons between 2 groups or > 2 groups, respectively. Multivariable regression was performed to confirm if any significant associations between adherence metrics and clinical characteristics and asthma outcomes identified from univariate analyses were still independent predictors after adjusting for the potential confounders of age, sex, baseline eosinophils and baseline lung function. Adjustment for baseline asthma questionnaire scores (AQLQ and ACT) was undertaken where the respective asthma questionnaire score was the outcome.

Statistical analysis was undertaken in R version 3·3^25^. Descriptive statistics are presented as proportions for categorical variables, means and standard deviations for normally distributed continuous variables, or medians and interquartile ranges otherwise.

## Results

### Participants

**Figure 4:**
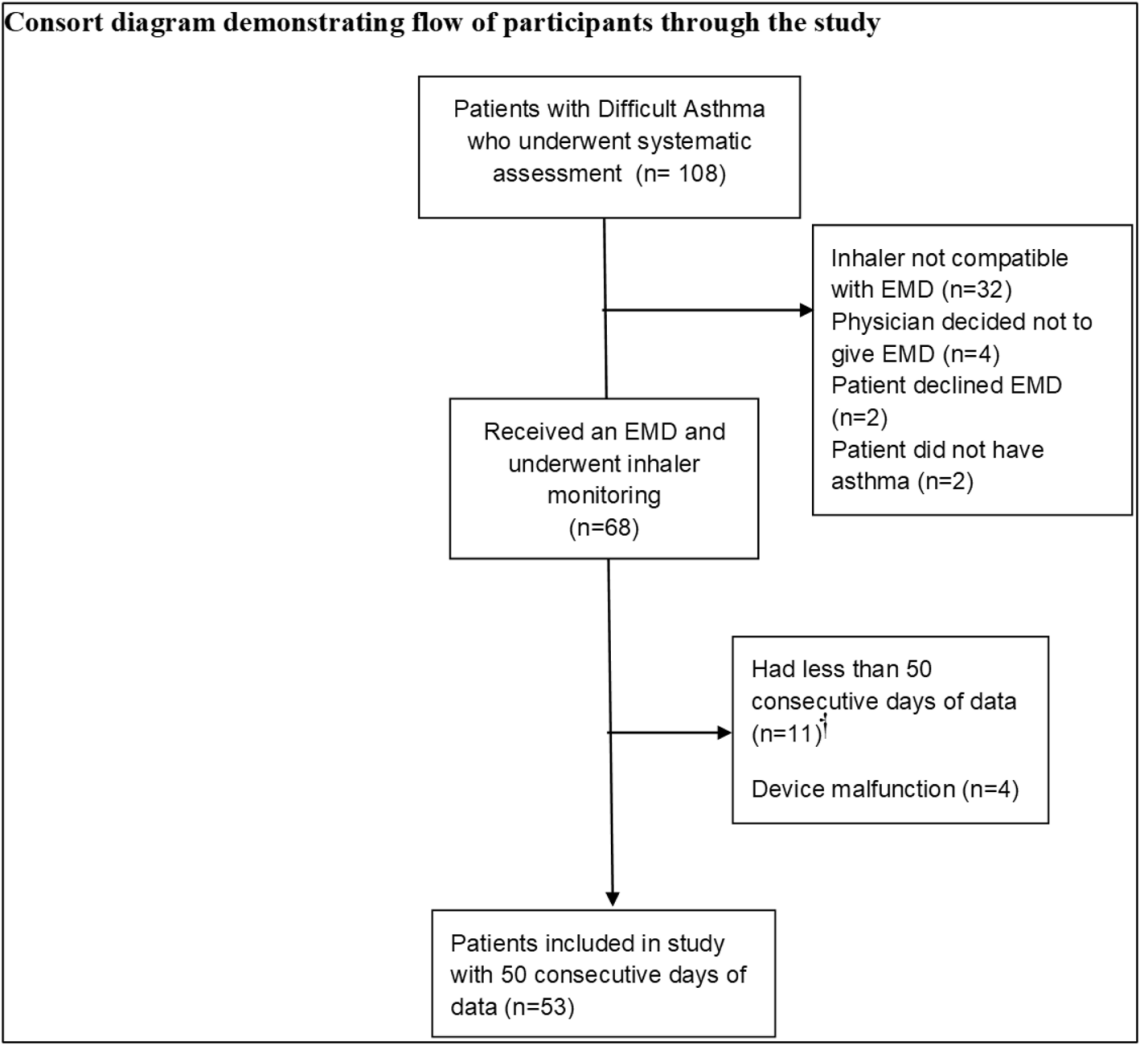
Consort diagram demonstrating flow of participants through the study. *EMD* electronic monitoring device. ^†^Due to EMD detachment. (Microsoft Powerpoint, version 2101, https://office.live.com/start/powerpoint.aspx).

Systematic assessment was undertaken by 108 patients. Forty (37%) did not receive a monitoring device: four (3.7%) from physician choice, two (1.9%) declined, two (1.9%) did not have asthma (and were diagnosed with vocal cord dysfunction), and 32 (29%) had inhalers with no compatible EMDs available in Australia. Among 68 patients who underwent inhaler monitoring, 11 (16.2%) devices had less than 50 days of data due to device detachment, and 4 (5.9%) devices malfunctioned (Fig. 4). These patients were excluded from the analysis. Of the 11 devices with missing data, the mean number of days monitored was 37, (SD 69, range 3–220 days).

Data were analysed from 53 patients (Table 1).

**Table 1:** Baseline patient characteristics.

	Total n = 53
**Demographics**	
Age years, mean (range, SD)	51 (19–77, 15)
Gender Female, n (%)	29 (55)
Body mass index kg/m^2^, mean (SD)	32 (8)
Smoking status	
Never	33 (62.3)
Ex-smoker	17 (32.1)
Current smoker	2 (3.8)
**Asthma medications, n(%)**	
Short acting muscarinic antagonist	4 (7.5)
Long acting beta agonist	1 (1.9%)
Inhaled corticosteroid	20 (37.7%)
Inhaled corticosteroid/long acting beta agonist combination	52 (98.1%)
Leukotriene receptor antagonist	10 (18.9%)
Long acting muscarinic antagonist	19 (35.8%)
Oral corticosteroids	10 (18.9%)
Theophylline	2 (3.8%)
Omalizumab (anti-IgE monoclonal antibody)	2 (3.8%)
Total number asthma medications, mean, range (SD)	2.3, 1–6 (1.2)
Total daily ICS dose mcg, mean, range (SD)	969, 200–2000 (475)
**Asthma severity**	
Pre-bronchodilator FEV_1_% predicted, mean (SD)	64 (21)
FEV_1_/FVC ratio	61 (16)
ACT score at visit one, median (IQR) (23)	11 (9–16·5)
AQLQ score at visit one, mean (SD) (24)	4 (1·2)
On high dose inhaled corticosteroids*, n(%)	44 (83)
**Asthma attack rate**	
Baseline attack number in the six months prior to visit one (mean, SD)	
Requiring oral corticosteroids	2.5 (2)
Requiring GP visit	2.4 (3.7)
Requiring ED presentation	0.8 (1.3)
Requiring hospital admission	0.4 (1)
Attack rate in the six months prior to visit three (mean, SD)	
Requiring oral corticosteroids	1.7 (2.9)
Requiring GP visit	2 (5.8)
Requiring ED presentation	0.4 (1)
Requiring hospital admission	0.3 (0.8)
**Asthma phenotype**	
FeNO result ppb, mean (range, SD)	35 (5–137, 32)
IgE kU/L, mean (range, SD)	528 (4–4304, 913)
Atopic (positive skin prick test or serum specific IgE to commonly tested aeroallergens), n(%)	37 (70)
Blood eosinophils × 10^9^/L, mean (range, SD)	0·37 (0–1·18, 0·31)

*FEV*_*1*:_Forced expiratory volume in one second, *FVC* forced vital capacity, *ACT* Asthma Control Test (scores range from 5 (poor asthma control) to 25 (complete asthma control), scores > 19 indicate well controlled asthma), *AQLQ* asthma quality of life questionnaire (out of 7, high score indicating better quality of life). *FeNO* fraction of expired nitric oxide, *IgE* immunoglobulin E, *GP* General practitioner, *ED* emergency department.

*Fluticasone propionate equivalent ≥ 500mcg daily.

### Adherence metrics and baseline clinical characteristics

Summary statistics of adherence metrics calculated for the first 50 days in all patients are reported in supplemental Table S1. No adherence metric was related to age, sex or baseline lung function on correlation testing (Supplemental Table S2). PT_mean,cap_ correlated with baseline asthma quality of life as measured by AQLQ (Spearman correlation, r_S_ = 0·284, p = 0·046). Large gaps in inhaler use (Gap_max_) and lower T-AUC were associated with a greater likelihood of previous intensive care or hospitalisation for an asthma attack in the six-month period prior to visit one (Wilcoxon rank sum test), Gap_max_: Z = − 2.068, p = 0·039 and Z = − 2.08, p = 0·037 respectively, T-AUC: Z = − 2.065, p = 0·039 and Z = − 2.042, p = 0·041 respectively).

Regarding entropy, higher H correlated negatively with baseline AQLQ and ACT scores (r_S_ = − 0·330, p = 0·019 and r_S_ = − 0·288, p = 0·041 respectively). Higher H_dec_ similarly correlated negatively with baseline AQLQ and ACT (r_S_ = -0·385, p = 0·006 and r_S_ = − 0·351, p = 0·012 respectively), and was further associated with higher SABA reliever use in terms of puffs and days per week (r_S_ = 0·318, p = 0·02 and r_S_ = 0·286, p = 0·04 respectively).

The relationships between entropy measures (H and H_dec_) and baseline ACT and reliever use remained significant following multivariable regression models adjusting for age, sex, baseline eosinophil count and baseline FEV, while all other measures did not (Table 2).

**Table 2:** Multivariable analysis relating adherence metrics to baseline clinical characteristics.

Baseline measures (adjusted for age, sex, peripheral blood eosinophils and FEV_1_)			
Adherence metric	Baseline measure	Coefficient [SE]	p value
Mean adherence (PT_mean,cap_)	AQLQ	0·19 [0.20]	0·36
Entropy (H)	ACT	**− 0·49 [0·17]**	**0·008**
	AQLQ	**− **0·29 [0·15]	0·065
Decreasing Entropy (H_dec_)	ACT	**− 0·51 [0·21]**	**0·026**
	AQLQ	**− **0·35 [0·19]	0·068
	Reliever use, puffs per week	**0·60 [0·28]**	**0·04**

Adherence metrics and baseline measures reported here are those which showed significant associations in univariate analyses.

*AQLQ* Asthma quality of life questionnaire, *ACT* asthma control test.

### Adherence metrics and subsequent outcomes at six months

**Figure 5:**
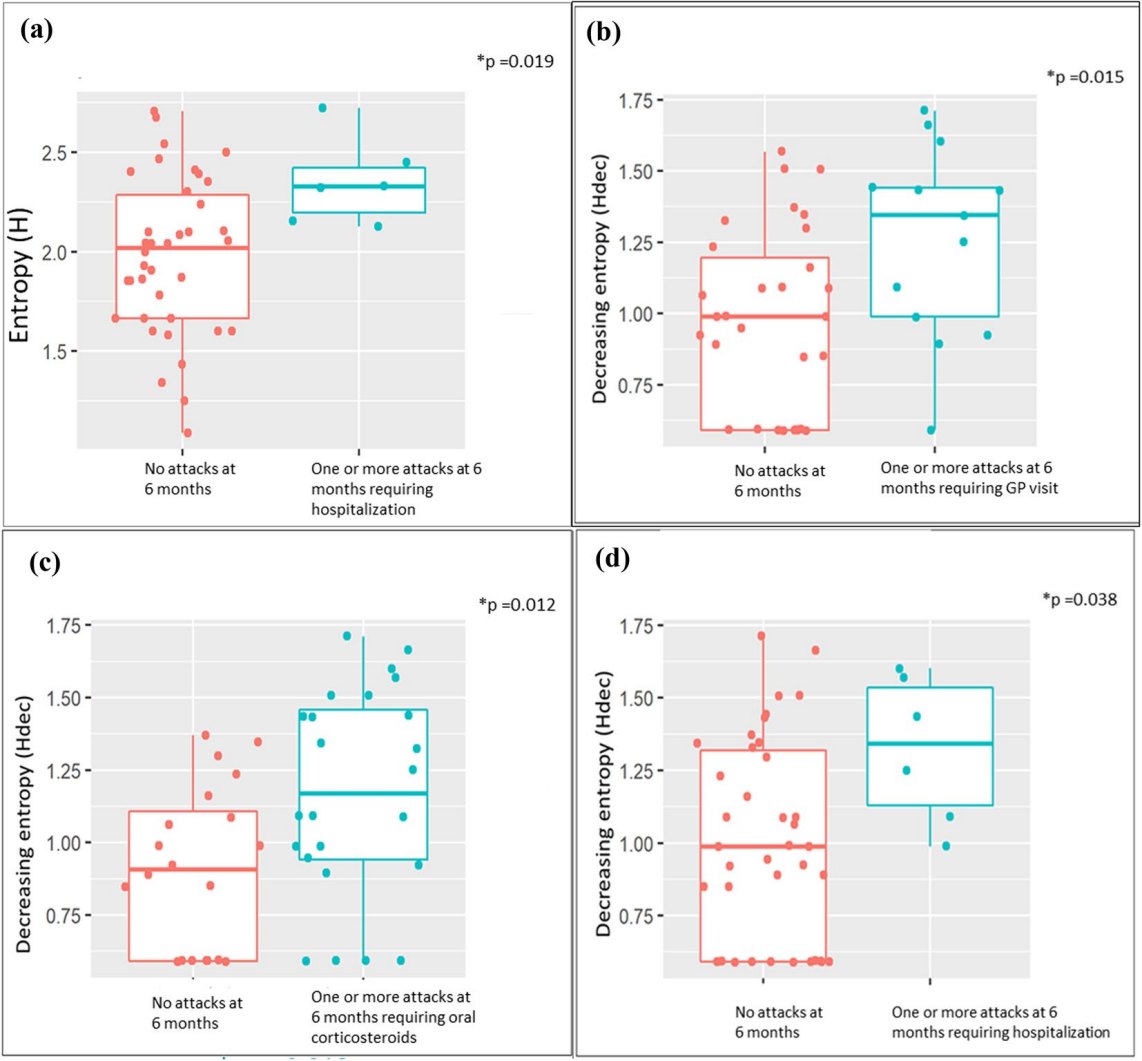
Entropy metrics (over day 0–50) predict asthma outcomes (over days 0–180). Panel (**a**): Entropy (H) and attacks requiring hospitalisation. Panels (**b**–**d**): Decreasing entropy (H_dec_) and attacks requiring general practitioner (GP) visit, oral corticosteroids or hospitalisation respectively. The boxes depict the 25th, 50th, and 75th percentiles while the whiskers depict the minimum and maximum values in the data. The individual data points are also shown as dots. (R version 3·3 https://www.R-project.org/).

Among all adherence metrics, only measures of entropy, measured in the first 50 days, were associated with asthma outcomes at six months (Supplemental Table S3). Higher H was associated with more asthma attacks requiring hospitalisation over six months prior to visit three (Z = − 2.34, p = 0·019, Fig. 5a).Higher H_dec_ was associated with more asthma attacks over the six months prior to visit three, requiring a visit to a general practitioner (Z = − 2.43, p = 0·015), oral corticosteroids (Z = − 2.508, p = 0·012), or hospitalisation (Z = − 2.07, p = 0·038, Fig. 5b–d). (All comparisons performed by Wilcoxon Rank Sums Test).

For regression analysis, it was only possible to adjust for one confounder at a time, due to the amount of patient data available at 6 months. Relationships between H and asthma attacks requiring hospitalisation remained significant regardless of adjustment for age, sex and baseline FEV_1_ (Table 3). Similarly, relationships between H_dec_ and attacks requiring oral corticosteroids or general practitioner visits also remained significant with these adjustments (Table 3) and also when adjusted for baseline number of attacks in the six months prior to visit one requiring oral corticosteroids (0.92, SE 0.38, p = 0.017), GP visit (1.06, SE 0.45, p = 0.018) or hospitalization (1.39, SE 0.68 p = 0.041). (Spearman coefficients reported).

**Table 3:** Multivariable analysis relating adherence metrics to 6 month outcomes.

Six-month outcomes (adjusted for age, sex, peripheral blood eosinophils and FEV_1_)					
Adherence metric	Outcome measure at 6 months	*Coefficient [SE] (p value) when adjusted for*			
		Age	Sex	Blood Eosinophils	FEV_1_
Entropy (H)	Attacks requiring hospitalisation	**1·35 [0·68] ** ** (p = 0·046)**	**1·34 [0·68] ** **(p = 0·047)**	2·37 [1·50] (p = 0·113)	**1·4 [0·69] ** **(p = 0·044)**
Decreasing Entropy (H_dec_)	Attacks requiring GP visit	**0·98 [0·49] ** **(p = 0·045)**	**0·98 [0·43] ** ** (p = 0·021)**	0·91 [0·48] (p = 0·059)	**1·03 [0·43] ** ** (p = 0·017)**
	Attacks requiring oral corticosteroids	**0·83 [0·39] ** ** (p = 0·034)**	**0·91 [0·38] ** ** (p = 0·017)**	0·50 [0·46] (p = 0·284)	**0·99 [0·40] ** ** (p = 0·014)**
	Attacks requiring hospitalisation	1·11 [0·60] (p = 0·064)	1·07 [0·59] (p = 0·068)	0·82 [0·69] (p = 0·231)	1·10 [0·58] (p = 0·058)

*GP* general practitioner. Adherence metrics and outcome measures reported here are those which showed significant associations in univariate analyses.

However, after adjustment for baseline eosinophil count, the relationships between H or H_dec_ and attacks at six months were no longer significant. Further examination showed that H (but not H_dec_) was correlated with baseline eosinophil count (r_S_ = 0·352, p = 0·045) suggesting collinearity between baseline eosinophils and H.

## Discussion

With increasing emphasis on inhaler adherence monitoring in airways diseases, particularly in the era of biologic therapies for severe asthma, there is a pressing need to identify the optimal metrics with which to measure inhaled controller adherence^7,26,27^. We showed that disordered controller use in difficult-to-treat asthma—as reflected by entropy analysis—reflected poor baseline asthma control and were associated with subsequent attacks of any severity. This could potentially be mediated through unchecked eosinophilic inflammation.

Entropy measures have previously been used to describe respiratory symptoms and breathing patterns, with higher entropy associated with adverse outcomes^13,28^. We designed entropy measures (H, H_dec_ and H_inc_) to measure the irregularity of day-to-day dose-taking behaviour, analogous to the original use of H in information theory to quantify the complexity in strings of text^29^. We used it to describe the diversity in patterns in observed transitions in adherence, choosing to also examine irregularity or diversity in increases and decreases in adherence, as they may be clinically relevant. In considering all (or a subset in the case of H_dec_ and H_inc_) of the elements in the transitional probability matrix, our method of calculating H differs from that of Usemann et al., where entropy was calculated from rows of elements and then averaged^13^. Nevertheless, H calculated using our method was highly correlated with their method when applied to this dataset (r = 0·912, p < 0·001, data available on request).

To accommodate the original study design, we chose a period of 50 days to maximise participants with sufficient data. This proof-of-concept study justifies validation in larger cohorts and the development of more dynamic measures of entropy, similar to our previous work on peak flows to predict attacks^30^.

Entropy metrics, specifically in relation to decreasing states (H_dec_) over a 50-day period, related to worse asthma control and increased short-acting reliever use at baseline. Notably, greater H_dec_ also predicted subsequent risk of attacks of any severity, whether requiring general practitioner visit, increase in oral steroids, or hospitalisation (the latter also predicted by H). That H_dec_, rather than H_inc_, has these relationships suggests that irregular drops in adherence may have more clinical impact than over-adherence. These relationships were no longer significant when adjusting for baseline peripheral eosinophil levels. The correlation between H (though not H_dec_) and peripheral eosinophils suggests that higher baseline eosinophil counts may represent previous poor adherence. We hypothesise that the same pattern of behaviour may then have continued during the period of monitoring, with unchecked eosinophilic inflammation subsequently leading to asthma attacks. Previous studies have demonstrated that peripheral eosinophils are an independent predictor for asthma attacks^31^. Within this small study, baseline blood eosinophils did not predict asthma attacks at 6 months, nor was FeNO related to any adherence measures (supplemental data).

While non-adherence can be intentional due to issues such as mistrust, lack of medication understanding, fixed beliefs and cost, unintentional disordered medication use may also indicate a corresponding degree of chaos in patients’ lives. In asthma, poor family routines accompanied diminished inhaler adherence in children^32^. In post-myocardial infarct patients, ‘life-chaos’—a highly variable daily routine with an inability to plan and anticipate the future, paralleled poor adherence to cardiac medication^15^. Similar life-chaos among patients with HIV was associated with increased health care use and missed clinic appointments^16^. We speculate that the extent of entropy in controller use in difficult-to-treat asthma may also reflect overall life-chaos. Measurement of entropy in inhaled controller use could be used in the clinic setting to target patients particularly with high H_dec_ for adherence interventions. Such patients may have otherwise been missed if conventional averaged adherence measures were used (Fig. 3). Entropy measures may also prompt the clinician to review the wider social situation of the patient for other indicators of ‘life chaos’.

As anticipated, conventional mean adherence (PT_mean,cap_) in our study (following adjustment for potential confounders) was not related to baseline asthma status, nor predicted longitudinal outcomes.

Similarly, neither variability in dosage nor timing metric was associated with clinically important outcomes (Supplemental tables S2 and S3). In a previous analysis of a clinical trial in moderate asthma, the use of AUC-based metrics *did* relate to asthma-related quality of life and lung function by peak flow measurement^10^. Note that our AUC metrics were based upon, but were not directly comparable to previously-published methods^10^, which accounted for technique/device errors using a specialised INCA device. Furthermore, our study cohort included consecutive patients drawn from clinical practice.

Our patient population had significant disease with poorly controlled symptoms and high exacerbation rates, despite having previously been assessed by respiratory specialists. We have previously demonstrated that this population still has high non-adherence rates despite specialist intervention. Our results are likely to be representative of difficult-to-treat asthma patients encountered in the ‘real world’, but may not represent less severe patients. The association of entropy with other behaviour that can affect adherence such as mistrust of medication, financial barriers, and not attending a pharmacy access to refill prescriptions would be worth pursuing with future research.

## Limitations

Given the complexity of difficult-to-treat asthma, poor disease control may relate to a wide range of disease and patient factors, e.g. biological severity, corticosteroid insensitivity, multimorbidity, poor self-management skills—all addressed in our clinic’s systematic protocol^33–35^. Notwithstanding the presence of such confounding issues, a significant effect of disordered controller use on risk of asthma attack remained detectable. However, it is possible our single-center study had insufficient statistical power from a reduction in data available due to device incompatibility device malfunction, missing data, small sample size and short duration of data collection, to detect weaker associations. We also relied on patient recollection for asthma attack history which could be inaccurate, although these data were verified when available in medical records. We explored a range of metrics, baseline characteristics, and asthma outcomes, so increasing the likelihood of a chance finding. However, the consistent pattern of results and their persistence following adjustment for confounding both support a true result. We only collected adherence data between visit one and two of our study (most consistently for 50 days), and analysed outcomes at day 180 (visit three). It is possible that adherence would have improved beyond 50 days, however we wished to analyse the impact of the patient’s initial adherence behaviour on future asthma outcomes. It is likely other aspects of adherence behaviour would add to the predictive power of entropy measures; larger validation datasets would enable further exploration as well as control for other possible confounders in the same model. Future studies could also explore the impact of patient socioeconomic status or device polypharmacy on entropy of inhaled controller use as well as examine aspects of ‘life chaos’ more qualitatively.

## Conclusions

We showed higher irregularity assessed by entropy in controller use of patients with difficult-to-treat asthma, with effects that appear mediated through eosinophilic inflammation, and were associated with an increased risk of future attacks. Entropy may reflect the ‘life chaos’ experienced by people with difficult-to-treat asthma, a possible target for appropriate intervention. Entropy analysis could guide future approaches to improve adherence and enhance patient health, potentially applicable to other domains of respiratory or other chronic disease.

## Supplementary Information

Supplementary Information.

## Abbreviations

ACT: Asthma control test
AQLQ: Asthma quality of life questionnaire
AUC: Area under the curve
EMD: Electronic monitoring device
FEV_1_: Forced expiratory volume in one second
FeNO: Fraction of exhaled nitric oxide
FVC: Forced vital capacity
GP: General practitioner
H: Entropy
H_dec_: Decreasing entropy states
H_inc_: Increasing entropy states
ICS: Inhaled corticosteroids
IgE: Immunoglobulin E
OCS: Oral corticosteroids
PT mean: Conventional mean adherence
SABA: Short acting bronchodilator

## References

[1] Barnes PJ (1998). Efficacy of inhaled corticosteroids in asthma. J. Allergy Clin. Immunol..

[2] Demarche SF (2017). Effectiveness of inhaled corticosteroids in real life on clinical outcomes, sputum cells and systemic inflammation in asthmatics: A retrospective cohort study in a secondary care centre. BMJ Open.

[3] GINA. Diagnosis and management of difficult-to-treat and severe asthma in adolescent and adult patients. (2019). Available online at https://ginasthma.org/wp-content/uploads/2018/11/GINA-SA-FINAL-wms.pdf.

[4] Vrijens B (2016). What we mean when we talk about adherence in respiratory medicine. J. Allergy Clin. Immunol. Pract..

[5] Kini V, Ho PM (2018). Interventions to improve medication adherence. JAMA.

[6] Gamble J, Stevenson M, McClean E, Heaney LG (2009). The prevalence of nonadherence in difficult asthma. Am. J. Respir. Crit. Care Med..

[7] Lee J (2018). Non-adherence in the era of severe asthma biologics and thermoplasty. Eur. Respir. J..

[8] Foster JM (2012). The reliability and patient acceptability of the SmartTrack device: A new electronic monitor and reminder device for metered dose inhalers. J. Asthma.

[9] Hew M, Reddel HK (2019). Integrated adherence monitoring for inhaler medications. JAMA.

[10] Sulaiman I (2016). A method to calculate adherence to inhaled therapy that reflects the changes in clinical features of asthma. Ann. Am. Thorac. Soc..

[11] Foster JM (2014). Inhaler reminders improve adherence with controller treatment in primary care patients with asthma. J. Allergy Clin. Immunol..

[12] Greene G (2018). A novel statistical method for assessing effective adherence to medication and calculating optimal drug dosages. PLoS ONE.

[13] Usemann J (2018). Dynamics of respiratory symptoms during infancy and associations with wheezing at school age. ERJ Open Res..

[14] Bravi A, Longtin A, Seely AJE (2011). Review and classification of variability analysis techniques with clinical applications. Biomed. Eng. Online.

[15] Zullig Leah L (2013). Association between perceived life chaos and medication adherence in a postmyocardial infarction population. Circ. Cardiovasc. Qual. Outcomes.

[16] Wong MD, Sarkisian CA, Davis C, Kinsler J, Cunningham WE (2007). The association between life chaos, health care use, and health status among HIV-infected persons. J. Gen. Intern. Med..

[17] Radhakrishna N (2016). Profile of difficult to treat asthma patients referred for systematic assessment. Respir. Med..

[18] Denton E (2020). Systematic assessment for difficult and severe asthma improves outcomes and halves oral corticosteroid burden independent of monoclonal biologic use. J. Allergy Clin. Immunol..

[19] Denton E (2018). Factors associated with dysfunctional breathing in patients with difficult to treat asthma. J. Allergy Clin. Immunol. Pract..

[20] Lee J (2020). Paradoxical vocal fold motion in difficult asthma is associated with dysfunctional breathing and preserved lung function. J. Allergy Clin. Immunol. Pract..

[21] Tay TR (2017). A structured approach to specialist-referred difficult asthma patients improves control of comorbidities and enhances asthma outcomes. J. Allergy Clin. Immunol. Pract..

[22] Denton E (2019). Severe asthma global evaluation (SAGE): An electronic platform for severe asthma. J. Allergy Clin. Immunol. Pract..

[23] Nathan RA (2004). Development of the asthma control test☆A survey for assessing asthma control. J. Allergy Clin. Immunol..

[24] Juniper EF (1992). Evaluation of impairment of health related quality of life in asthma: Development of a questionnaire for use in clinical trials. Thorax.

[25] The R Project for Statistical Computing (2017).

[26] Costello RW, Cushen B (2020). Looking back to go forward: adherence to inhaled therapy before biologic therapy in severe asthma. Eur. Respir. J..

[27] Ancona G (2020). Adherence to corticosteroids and clinical outcomes in mepolizumab therapy for severe asthma. Eur. Respir. J..

[28] Engoren M (1998). Approximate entropy of respiratory rate and tidal volume during weaning from mechanical ventilation. Crit. Care Med..

[29] Shannon CE (1948). A mathematical theory of communication. Bell Syst. Tech. J..

[30] Thamrin C (2011). Predicting future risk of asthma exacerbations using individual conditional probabilities. J. Allergy Clin. Immunol..

[31] Zeiger RS (2014). High blood eosinophil count is a risk factor for future asthma exacerbations in adult persistent asthma. J. Allergy Clin. Immunol. Pract..

[32] Fiese BH, Wamboldt FS, Anbar RD (2005). Family asthma management routines: Connections to medical adherence and quality of life. J. Pediatr..

[33] Hew M (2006). Relative corticosteroid insensitivity of peripheral blood mononuclear cells in severe asthma. Am. J. Respir. Crit. Care Med..

[34] Hew M, Heaney LG, Chung KF, Israel E, Gibson PG (2019). Severe Asthma [ERS Monograph].

[35] Hew M (2020). Systematic assessment of difficult-to-treat asthma: Principles and Perspectives. J. Allergy Clin. Immunol. Pract..

