# Supplementary material for: Dynamics of inhaled corticosteroid use are associated with asthma attacks
Source: Sci Rep. 2021 Jul 19;11:14715. doi: 10.1038/s41598-021-94219-z (PMC8289909; doi:10.1038/s41598-021-94219-z)
Supplement: Supplementary file 1 — Supplementary Information. [file 41598_2021_94219_MOESM1_ESM.docx]

**SUPPLEMENTARY INFORMATION**

**Dynamics of inhaled corticosteroid use are associated with asthma attacks**

**Authors**

Joy Lee BHB MBChB FRACP*^1,2^, Jacqueline Huvanandana BEng PhD^3^, Juliet M. Foster BSc (Hons) PhD^3^, Helen K. Reddel MBBS FRACP PhD^3^, Michael J. Abramson FRACP FAFPHM PhD^1,2^, Cindy Thamrin PhD^⸶3^, Mark Hew PhD MSc FRACP^⸶1,2^

^⸶^*These authors contributed equally to this work.*

**Table S1.** Summary statistics of adherence metrics calculated for the first 50 days in all patients

| **Adherence metric** | **Median (25^th^ centile, 75^th^ centile)** |
| --- | --- |
| PT_mean_,_cap_, % | 88 (66.5, 93) |
| PT_mean_, % | 92 (72, 101) |
| PT_SD_, % | 34.5 (25.9, 44.8) |
| PT_CV_, % | 40.7 (29.4, 57.7) |
| Gap_max_, days | 1 (0, 4) |
| Entropy (H) | 2.04 (1.66, 2.32) |
| Increasing Entropy (H_inc_) | 1.09 (0.95, 1.16) |
| Decreasing Entropy (H_dec_) | 1.09 (0.59, 1.37) |
| T-AUC, % | -1.76 (-8.16, 0.00) |
| D-AUC, % | -7.36 (-26.6, 2.26) |
| Prod-AUC, % | -9.45 (-34.37, 0.08) |

Please see main text for adherence metrics abbreviations

**Table S2:** **Baseline Univariate Analyses**

| **Variable** | **PT_mean,cap_** | **PT_mean_** | **PT_SD_** | **PT_CV_** | **Gap_max_** | **H** | **H_inc_** | **H_dec_** | **T-AUC** | **D-AUC** | **Prod-AUC** |
| --- | --- | --- | --- | --- | --- | --- | --- | --- | --- | --- | --- |
| Sex (male)⸶ | -1.47(0.14) | -1.35(0.18) | -0.49(0.62) | -1.06(0.29) | -0.48(0.63) | -0.61(0.54) | -0.63(0.53) | -0.57(0.57) | -0.53(0.60) | -1.15(0.25) | -1.28(0.20) |
| Age (years) ⸶⸶ | -0.056(0.69) | -0.072(0.61) | -0.018(0.90) | -0.008(0.96) | 0.072(0.61) | -0.074(0.60) | -0.018(0.9) | -0.212(0.13) | -0.039(0.78) | -0.034(0.81) | -0.02(0.89) |
| FEV_1_ (litres) ⸶⸶ | 0.13(0.37) | 0.19(0.18) | -0.015(0.92) | -0.104(0.46) | -0.077(0.58) | -0.099(0.48) | 0.091(0.52) | -0.116(0.41) | 0.079(0.57) | 0.153(0.27) | 0.15(0.29) |
| Eosinophils (x10^9^/L) ⸶⸶ | -0.31(0.08) | -0.31(0.08) | -0.036(0.84) | 0.24(0.17) | 0.22(0.21) | 0.352(0.045*) | 0.291(0.1) | 0.109(0.547) | -0.29(0.1) | -0.25(0.169) | -0.23(0.21) |
| Reliever use (puffs per week) ⸶⸶ | -0.133(0.35) | -0.213(0.13) | -0.279(0.045*) | -0.169(0.232) | -0.224(0.11) | 0.214(0.13) | -0.175(0.22) | 0.336(0.015*) | 0.20(0.15) | -0.25(0.07) | -0.23(0.11) |
| ACT score⸶⸶ | 0.155(0.28) | 0.16(0.26) | 0.114(0.42) | 0.078(0.59) | 0.113(0.43) | -0.288(0.041*) | 0.112(0.44) | -0.351(0.012*) | -0.064(0.66) | 0.17(0.24) | 0.16(0.25) |
| AQLQ score⸶⸶ | 0.284(0.046*) | 0.247(0.08) | 0.006(0.97) | -0.146(0.31) | -0.068(0.64) | -0.33(0.019*) | -0.012(0.93) | -0.385(0.006**) | 0.116(0.422) | 0.26(0.07) | 0.25(0.08) |

⸶Analysis – Wilcoxon Rank Sums, ⸶⸶Analysis – Spearman correlation. Z statistic or Spearman correlation coefficient shown, with P values in brackets.

*significant to p<0.05, **significant to p<0.005

Reliever use -daytime average of short acting beta agonist over seven-day period, Eosinophils – peripheral blood sample, FEV_1_ – Forced expiratory volume in one second, ACT – asthma control test, AQLQ – asthma quality of life questionnaire

Please see main text for adherence metrics abbreviations

**Table S3: 6-month outcomes univariate analyses**

| **Exacerbations in 6 months** | **PT_mean,cap_** | **PT_mean_** | **PT_SD_** | **PT_CV_** | **Gap_max_** | **H** | **H_inc_** | **H_dec_** | **T-AUC** | **D-AUC** | **Prod-AUC** |
| --- | --- | --- | --- | --- | --- | --- | --- | --- | --- | --- | --- |
| Requiring OCS | -0.08  (0.93) | -0.34  (0.73) | -1.24  (0.22) | -0.81  (0.42) | -1.72  (0.09) | -1.33  (0.18) | -1.46  (0.15) | -2.51  (0.012*) | -1.53  (0.13) | -0.34  (0.73) | -0.13  (0.90) |
| Requiring GP visit | -0.59  (0.55) | -0.57  (0.57) | -0.03  (0.98) | -0.46  (0.64) | -0.61  (0.54) | -0.58  (0.56) | -0.18  (0.86) | -2.43  (0.015*) | -0.32  (0.75) | -0.57  (0.57) | -0.54  (0.59) |
| Requiring ED visit | -1.39  (0.17) | -1.81  (0.07) | -0.41  (0.68) | -0.53  (0.59) | -0.08  (0.94) | -1.13  (0.26) | -0.18  (0.86) | -0.71  (0.48) | -0.11  (0.91) | -1.81  (0.07) | -1.87  (0.061) |
| Requiring hospitalisation | -1.23  (0.22) | -1.56  (0.12) | -0.26  (0.80) | -0.46  (0.64) | -0.07  (0.94) | -2.34  (0.019*) | -0.77  (0.44) | -2.07  (0.038*) | -0.087  (0.93) | -1.52  (0.13) | -1.52  (0.13) |

All analyses completed via Wilcoxon Rank Sum. Z statistic shown, with P values in brackets. OCS – oral corticosteroid course or increase in steroid dose from baseline, GP – general practitioner, ED- emergency department. *significant to p<0.05 level
